# Supplementary material for: Heat adaptation of phage T7 under an extended genetic code
Source: Virus Evol. 2021 Dec 1;7(2):veab100. doi: 10.1093/ve/veab100 (PMC8923235; doi:10.1093/ve/veab100)
Supplement: veab100_Supp [file veab100_supp.zip › Sequencing_Analysis.zip!/Sequencing_Analysis/A1/evidence/JC_63.html]

BRESEQ :: Evidence


| New junction evidence | | | | | | | | | | | |
| --- | --- | --- | --- | --- | --- | --- | --- | --- | --- | --- | --- |
|  | | seq id | position | reads (cov) | reads (cov) | score | skew | freq | annotation | gene | product |
| \* | ? | NC\_001604.1 | = 16604 | 2883 (0.930) | 10 (0.000) | 9/590 | 41.6 | 0.35% | intergenic (–/–) | *–/–* | –/– |
|  | ? | NC\_001604.1 | 17028 = | 2874 (0.950) |  |  |  |  | intergenic (–/–) | *–/–* | –/– |
| Rejected: Coverage evenness skew score above cutoff. | | | | | | | | | | | |
| Rejected: Frequency below/above cutoff threshold. | | | | | | | | | | | |

Only 100 of 242 total aligned reads displayed.  

`GGCATGGGTACATGATGAAATCCAAGTAGGCTGCCGTACCGAAGAGATTGCTCAGGTGGTCATTGAGACCGCACAAGAAGCGATGCGCTGGGTTGGAGACCACTGGAACTTCCGGTGTCTTCTGGATACCGAAGGTAAGATGGGTCCTAATTGGGCGATTTGCCACTGATACAGGAGGCTACTCATGAACGAAAGACACTTAACAGGTGCTGCTTCTGAAATGCTAGTAGCCTACAAATTTACCAAAGCTGGGTACACTGTCTATTACCCTATGCTGACTCAGAGTAAAGAGGACTTGGTTGTATG‑‑‑‑‑‑‑‑‑‑‑‑‑‑‑‑‑‑‑‑‑‑‑‑‑‑‑‑‑‑‑‑‑‑‑‑‑‑‑‑‑‑‑‑‑‑‑‑‑‑‑‑‑‑‑‑‑‑‑‑‑‑‑‑‑‑‑‑‑‑‑‑‑‑‑‑‑‑‑‑‑‑‑‑‑‑‑‑‑‑‑‑‑‑‑‑‑‑‑‑‑‑‑‑‑‑‑‑‑‑‑‑‑‑‑‑‑‑‑‑‑‑‑‑‑‑‑‑‑‑‑‑‑‑‑‑‑‑‑‑‑‑‑‑‑‑‑‑‑‑‑‑‑‑‑‑‑‑‑‑‑‑‑‑‑‑‑‑‑‑‑‑‑‑‑‑‑‑‑‑‑‑‑‑‑‑‑‑‑‑‑‑‑‑‑‑‑‑‑‑‑‑‑‑‑‑‑‑‑‑‑‑‑‑‑‑‑‑‑‑‑‑‑‑‑‑‑‑‑‑‑‑‑‑‑‑‑‑‑‑‑‑‑‑‑‑‑‑‑‑‑‑‑‑‑‑‑‑‑‑‑‑‑‑‑‑‑‑‑‑‑‑‑‑‑‑‑‑‑‑‑‑‑‑‑‑‑‑‑‑‑‑‑‑‑‑‑‑‑‑  >  NC_001604.1/16299‑16604`  
`‑‑‑‑‑‑‑‑‑‑‑‑‑‑‑‑‑‑‑‑‑‑‑‑‑‑‑‑‑‑‑‑‑‑‑‑‑‑‑‑‑‑‑‑‑‑‑‑‑‑‑‑‑‑‑‑‑‑‑‑‑‑‑‑‑‑‑‑‑‑‑‑‑‑‑‑‑‑‑‑‑‑‑‑‑‑‑‑‑‑‑‑‑‑‑‑‑‑‑‑‑‑‑‑‑‑‑‑‑‑‑‑‑‑‑‑‑‑‑‑‑‑‑‑‑‑‑‑‑‑‑‑‑‑‑‑‑‑‑‑‑‑‑‑‑‑‑‑‑‑‑‑‑‑‑‑‑‑‑‑‑‑‑‑‑‑‑‑‑‑‑‑‑‑‑‑‑‑‑‑‑‑‑‑‑‑‑‑‑‑‑‑‑‑‑‑‑‑‑‑‑‑‑‑‑‑‑‑‑‑‑‑‑‑‑‑‑‑‑‑‑‑‑‑‑‑‑‑‑‑‑‑‑‑‑‑‑‑‑‑‑‑‑‑‑‑‑‑‑‑‑‑‑‑‑‑‑‑‑‑‑‑‑‑‑‑‑‑‑‑‑‑‑‑‑‑‑‑‑‑‑‑‑‑‑‑‑‑‑‑‑‑‑‑‑‑‑‑‑‑‑gtatgGAAGGATTGATGACATTCGTAGTACGTACATCATTTCGTGAGGCCATTAAGGACATGCACGAAGAGTATGCAGATAAGGACTCTTTCAAACAATCTCCTGCAACAGTACGGGAGGTGTTCTGATGTCTGACTACCTGAAAGTGCTGCAAGCAATCAAAAGTTGCCCTAAGACTTTCCAGTCCAACTATGTACGGAACAATGCGAGCCTCGTAGCGGAGGCCGCTTCCCGTGGTCACATCTCGTGCCTGACTACTAGTGGACGTAACGGTGGCGCTTGGGAAATCACTGCTTCCGGTACTC  >  NC_001604.1/17028‑17327`  

                                                                                                                                                                                                                                                                                                                                                                                                                                                                                                                                                                                                                                
 `gaATGGGTACATGATGAAATCCAAGTAGGCTGCCGTACCGAAGAGATTGCTCAGGTGGTCATTGAGACCGCACAAGAAGCGATGCGCTGGGTTGGAGACCACTGGAACTTCCGGTGTCTTCTGGATACCGAAGGTAAGATGGGTCCTAATTGGGCGATTTGCCACTGATACAGGAGGCTACTCATGAACGAAAGACACTTAACAGGTGCTGCTTCTGAAATGCTAGTAGCCTACAAATTTACCAAAGCTGGGTACACTGTCTATTACCCTATGCTGACTCAGAGTAAAGAGGACTTGGTTG                                                                                                                                                                                                                                                                                                                  <  1:474771/299‑1`  
 `GCATGGGTACATGATGAAATCCAAGTAGGCTGCCGTACCGAAGAGATTGCTCAGGTGGTCATTGAGACCGCACAAGAAGCGATGCGCTGGGTTGGAGACCACTGGAACTTCCGGTGTATTCTGGATACCGAAGGTAAGATGGGTCCTAATTGGGCGATTTGCCACTGATACAGGAGGCTACTCATGAACGAAAGACACTTAACAGGTGCTGCTTCTGAAATGCTAGTAGCCTACAAATTTACCAAAGCTGGGTACACTGTCTATTACCCTATGCTGACTCAGAGTAAAGAGGACTTGGTTG                                                                                                                                                                                                                                                                                                                  >  1:378796/1‑301`  
 `GCATGGGTACATGATGAAATCCAAGTAGGCTGCCGTACCGAAGAGATTGCTCAGGTGGTCATTGAGACCGCAAAAGAAGCGATGCGCTGGGTTGGAGACCACTGGAACTTCCGGTGTCTTCTGGATACCGAAGGTAAGATGGGTCCTAATTGGGCGATTTGCCACTGATACAGGAGGCTCCTCATGAACGAAAGACACTTAACAGGTGCTGCTTCTGAAATGCTAGTAGCCTACAAATTTACCAAAGCTGGGTACACTGTCTATTACCCTATGCTGACTCAGAGTAAAGAGGACTTGGTTG                                                                                                                                                                                                                                                                                                                  >  1:157020/1‑301`  
  `CATGGGTACATGATGAAATCCAAGTAGGCTGCCGTACCGAAGAGATTGCTCAGGTGGTCATTGAGACCGCACAAGAAGCGATGCGCTGGGTTGGAGACCACTGTAACTTCCGGTGTCTTCTGGATACCGAAGGTAAGCTGGGTCCTAATTTGGCGATTTGCCACTGATACAGGAGGCTACTCATGAACGAAAGACACTTAACAGGTGCTGCTTCTGAAATGCTAGTAGCCTACAAATTTACCAAAGCTGGGTACACTGTCTATTACCCTATGCTGACTCAGAGTAAAGAGGACTTGGTTGT                                                                                                                                                                                                                                                                                                                 >  1:236568/1‑301`  
   `ATGGGTACATGAAGAAATCCAAGGAGGCTGCCGTACCGAAGAGATTGCTCAGGTGGTCAATGAGACCGCACAAGAAGCGATGCGCTGGGTTGGAGACCGCTGGAACTTCCCGTGTCTTCTGGATACCGAAGGTAAGATGGGTCCTAATTGGGCGATTTGCCACTGATACAGGAGGCTACTCATGAACGAAAGACACTTAACAGGTGCTGCTTCTGAAATGCTAGTAGCCTACAAATTTGCCAAAGCTGGGTACACTGTCTATTACCATATGCTGACTCAGAGTAAAGAGGACTTGGTTGTA                                                                                                                                                                                                                                                                                                                <  1:100111/301‑1`  
    `agggacagTGATGAAATCCAAGTAGGCTGCCGTACCGAAGAGATTGCTCAGGTGGTCATTGAGACCGCACAAGAAGCGATGCGCTGGGTTGGAGACCACTGGAACTTCCGGTGTCTTCTTGATACCGAAGGTAAGATGGGTCCTAATTGGGCGATTTGCCACTGATACAGGAGGCTACTCATGAACGAAAGACACTTAACAGGTGCTGCTTCTGAAATGCTAGTAGCCTACAAATTTACCAAAGCTGGGTACACTGTCTATTACCCTATGCTGACTCAGAGTAAAGAGGACTTGGTTGTAT                                                                                                                                                                                                                                                                                                               <  1:263154/293‑1`  
    `TGGGTACATGATGAAATCCAAGTAGGCTGCCGTACCGAAGAGATTGCTCAGGTGGTCATTGAGACCGCACAAGAAGCGATGCGCTGGGTTGGAGACCACTGGAACTTCCGGTGTCTTCTGGATACCGAAGGTAAGATGGGTCCTAATTGGGCGATTTGCCACTGATACAGGAGGCTACTCATGAACGAAAGACACTTAACAGGTGCTGCTTCTGAAATGCTAGTAGCCTACAAATTTACCAAAGCTGGGTACACTGTCTATTACCCTATGCTGACTCAGAGTAAAGCGGACTTGGTTGTAT                                                                                                                                                                                                                                                                                                               >  1:119024/1‑301`  
     `agagcaaatggttgggtaagGTATGCTGCCGTACCGAAGAGATGGCTCAGGTGATAATTGAGACAGAATAAGAATCTATAAGCTGGGTTGGAGAGCAGTGGATCTTCCGGTGTCTTCTGGATACTGAAGGTAAGATGGGTCCTAATTGGGCGATTTGCAACTGATACAGGAGGCTACTCATGAACGAAAGACACTTAAAAGGTGATTCTACTGAAATGCTAGTAGCCTACAAATTTACCAAAGCTGGGTACACTGTCTATTACCCTATGCTGACTCAGAGTAAAGAGGACTTGGTTGTATG                                                                                                                                                                                                                                                                                                              <  1:502364/281‑1`  
     `agaagagacagGAAATCCTAGTAGGCTGCCGTACCGAAGAGATTGCTCAGGTGGTCATTGAGACCGCACAAGAAGCGATGCGCTGGTTTGGAGACCACTGGAACTTCCGGTGTCTTCTGGATACCGAAGGTAAGATGGGTCCTAATTGGGCGATTTGCCACTGATACAGGAGGCTACTCATGAACGAAAGACACTTAACAGGTGCTGCTTCTGAAATGCTAGTAGCCTACAAATTTACCAAAGCTGGGTACACTGTCTATTACCCTATGCTGACTCAGAGTAAAGAGGACTTGGTTGTATG                                                                                                                                                                                                                                                                                                              <  1:164983/290‑1`  
     `GGGTACCTGATGAAATCCAAGTAGGCTGCCGTACCGAAGAGATTGCTCAGGTGGTCATGGAGACCGCACAAGAAGCGATGCGCTGGGTTGGAGACCACTGGAACTTCCGGTGTCTTCTGGATACCGAAGGTAAGATGGGTCCTAATTGGGCGATTTGCCACTGATACAGGAGGCTACTCATGAACGAAAGACACTTAACAGGTGCTGCTTCTGAAATGCTAGTAGCCTACAAATTTACCAAAGCTGGGTACACTGTCTATTACCCTATGCTGACTCAGAGTAAAGAGGACTTGGTTGTATG                                                                                                                                                                                                                                                                                                              <  1:42775/301‑1`  
     `GGGTACATGATGAAATCCAAGTAGGCTGCCGTACCGAATGGATTGCTCAGGTGGTCATTGAGACCGCACAAGAAGCGATGCGCTGGGTTGGAGACCACTGGAACTTCCGGTGTCTTCTGGATACCGAAGGTAAGATGGGTCCGAATTGGGCGATTTGCCACTGATACAGGAGGCTACTCATGAAAGAAAGACACTTAACAGGTGCTGCTTCTGAAATGCTAGTAGCCTACAAATTTACCAAAGCTGGGTACACTGTCTATTACCCTATGCTGACTCAGAGTAAAGAGGACTTGGTTGTATG                                                                                                                                                                                                                                                                                                              <  1:316267/301‑1`  
     `GGGTACATGATGAAATCCAAGTAGGCTGCCGTACCGAAGAGATTGCTCAGGTGGTCATTTAGACCGCACAAGAAGCGATGCGCTGGGTTGGATACCACTGGAACTTCCGGTGTCTTCTGGATACCGAAGGTAAGATGGGTCCTAATTGGGCGATTTGCCACTGATACAGGAGGCTACTCATGAACGAAAGACACTTAACAGGTGCTGCTTCTGAAATGCTAGTAGCCTACAAATTTACCAAAGCTGGGTACACTGTCTATTACCCTATGCTGACTCAGAGTAAAGAGGACTTGGTTGTATt                                                                                                                                                                                                                                                                                                              >  1:192105/1‑300`  
     `GGGTACATGATGAAATCCAAGTAGGCTGCCGTACCGAAGAGATTGCTCAGGTGGTCATTGAGCCCGCACAAGAAGCGATGCGCTGGGTTGGAGACCACTGGAACTTCCGGTGTCTTCTGGATACCGCAGGTAAGATGGGTCCTAATTGGGCTATTTGCCACTGATACTGGAGGCTATTCATGAACGAAAGACACTTAACAGGTGCTCCTTCTGAAATGCTAATAGCCTACAAATTTACCAAAGCTGGGTACTCTGTCTCTTACCCTATGCTGACTCAGAGTAAAGAGGACTTGGTTGTATG                                                                                                                                                                                                                                                                                                              >  1:466224/1‑301`  
     `GGGTACATGATGAAATCCAAGTAGGCTGCCGTACCGAAGAGATTGCTCAGGTGGTCATTGAGACCGCACAAGAAGCGATGCGCTGGGTTGGAGGCCACTGGAACTTCCGGTGTCTTCTGGATACCGAAGGTAAGATGGGTCCTAATTGGGCGATTTGCCACTGATACATGAGGCTACTCATGAACGAAAGACACTTAACAGGTGCTGCTTCTGAAATGCTAGTAGCCTACAAATTTACCAAAGCTGGGTACACTGTCTATTACCCTATGCTGACTCAGAGTAAAGAGGACTTGGTTGGATG                                                                                                                                                                                                                                                                                                              <  1:480463/301‑1`  
     `GGGTACATGATGAAATCCAAGTAGGCTGCCGTACCGAAGAGATTGCTCAGGTGGTCATTGAGACCGCACAAGAAGCGATGCGCTGGGTTGGAGACCACTGGAACTTCCGGTGTCTTCTGGATACCGAAGGTAAGATGGGTCCTACTTGGGCGATTTGCCACTGATACAGGAGGCTACTCATGAACGAAAGACACTTAACAGGTGCTGCTTCTGAAATGCTAGTAGCCTACAAATTTACCAAAGCTGGGTACACTGTCTATTACCCTATGCTGACTCAGAGTAAAGAGGACTTGGTTGTATG                                                                                                                                                                                                                                                                                                              >  1:390863/1‑301`  
     `GGGTACATGATGAAATCCAAGTAGGCTGCCGTACCGAAGAGATTGCTCAGGTGGTCATTGAGACCGCACAAGAAGCGATGCGCTGGGTTGGAGACCACTGGAACTTCCGGTGTCTTCTGGATACCGAAGGTAAGATGGGTCCTAATTTGGCGATTTGCCACTGATACAGGAGGCTACTCATGAACGAAAGACACTTAACAGGTGCTGCTTCTGAAATGCTAGTAGCCTACAAATTTACCAAAGCTGGGTACACTGTCTATTACCCTATGCTGACTCAGAGTAAAGAGGACTTGGTTGctgt                                                                                                                                                                                                                                                                                                              >  1:34450/1‑297`  
     `GGGTACATGATGAAATCCAAGTAGGCTGCCGTACCGAAGAGATTGCTCAGGTGGTCATTGAGACCGCACAAGAAGCGATGCGCTGGGTTGGAGACCACTGGAACTTCCGGTGTCTTCTGGATACCGAAGGTAAGATGGGTCCTAATTGGGCGATTTGCCACTGATACAGGAGGCTACTCATGAACGACAGACACTTAACAGGTGCTGCTTCTGAAATGCTAGTAGCCTACAAATTTACCAAAGCTGGGTACACTGTCTATTAGCCTATGCTGTCTCAGAGTAAAGAGGACTTGGTTGTATG                                                                                                                                                                                                                                                                                                              >  1:58836/1‑301`  
     `GGGTACATGATGAAATCCAAGTAGGCTGCCGTACCGAAGAGATTGCTCAGGTGGTCATTGAGACCGCACAAGAAGCGATGCGCTGGGTTGGAGACCACTGGAACTTCCGGTGTCTTCTGGATACCGAAGGTAAGATGGGTCCTAATTGGGCGATTTGCCACTGATACAGGAGGCTACTCATGAACGAAAGACACTTAACAGGTGCTGCTTCTGAAATGCTAGTAGCCTACAAATTTACCAAAGCTTGGTACACTGTCTATTACCCTATGCTGACTCAGAGTAAAGAGGACTTGGTTGTATt                                                                                                                                                                                                                                                                                                              >  1:123613/1‑300`  
     `GGGTACATGATGAAATCCAAGTAGGCTGCCGTACCGAAGAGATTGCTCAGGTGGTCATTGAGACCGCACAAGAAGCGATGCGCTGGGTTGGAGACCACTGGAACTTCCGGTGTCTTCTGGATACCGAAGGTAAGATGGGTCCTAATTGGGCGATTTGCCACTGATACAGGAGGCTACTCATGAACGAAAGACACTTAACAGGTGCTGCTTCTGAAATGCTAGTAGCCTACAAATTTACCAAAGCTGGGTACACTGTCTATTACCCTATGCTGACTCAGAGTAAAGAGGACTTGGTTGTATt                                                                                                                                                                                                                                                                                                              >  1:151405/1‑300`  
     `GGGTACATGATGAAATCCAAGTAGGCTGCCGTACCGAAGAGATTGCTCAGGTGGTCATTGAGACCGCACAAGAAGCGATGCGCTGGGTTGGAGACCACTGGAACTTCCGGTGTCTTCTGGATACCGAAGGTAAGATGGGTCCTAATTGGGCGATTTGCAACTGATACAGGAGGCTACTCATGAACGAAAGACACTTAACAGGTGCTGCTTCTGAACTGCTAGTAGCCTACAAATTTACCAAAGCTGGGTACACTGTCTATTACCCTATGCTGACTCAGAGTAAAGAGGACTTGGTTGctgt                                                                                                                                                                                                                                                                                                              >  1:108829/1‑297`  
     `GGGTACATGATGAAATCCAAGTAGGCTGCCGTACCGAAGAGATTGCTCAGGTGGTCATTGAGACCGCACAAGAAGCGATGCGCTGGGTTGGAGACCACTGGAACTTCCGGAGTCTTCTGGATACCGAAGATAAGATGGGTCCTAATTGGGCGATTTGCCACTGATACAGGAGGCTACTCATGAACGAAAGACACTTAACAGGTGCTGCTTCTGAAATGCTAGTAGCCTACAAATTTACCAAAGCTGGGTACACTGCCTATTACCCTATGCTGACTCAGAGTAAAGAGGACTTGGTTGTATG                                                                                                                                                                                                                                                                                                              >  1:132451/1‑301`  
     `GGGTACATGATGAAATCCAAGTAGGCTGCCGTACCGAAGAGATTGCTCAGGTGGTCATTGAGACCGAACAAGAAGCGATGCGCTGGGTTGGAGACCACTGGAACTTCCGGTGTCTTCTGGATACCGAAGGTAAGATGGGTCCTAATTGGGCGATTTGCCACTGATACAGGAGGCTACTCATGAACGAAAGACACTTAACAGGTGCTGCTTCTGAAATGCTAGTAGCCTACAAATTTACCAAAGCTGGGTACACTGTCTATTACCCTATGCTGACTCAGAGTAAAGAGGACTTGGTTGTATG                                                                                                                                                                                                                                                                                                              <  1:89101/301‑1`  
     `GGGTACATGATGAAATCCAAGTAGGCTGCCGTACCGAAGAGATTGATCAGGTGGTCATTGAGACCGCACAAGAAGCGATGCGCTGGGTTGGAGACAACTGGAACTTCCGGTGTCTTCTGGATACCGAAGGTAAGATGGGTCCTAATTGGGCGATTTGCCACTGATACAGGAGGCTACTCATGAACGAAAGACACTTAACAGGTGCTGCTTCTGAAATGCTAGTAGCCTACAAATTTACCAAAGCTGGGTACACTGTCTATTACCCTATGCTGACTCAGAGTAAAGAGGACTTGGTTGTATG                                                                                                                                                                                                                                                                                                              <  1:443520/301‑1`  
     `GGGTACATGATGAAATCCAAGTAGGCTGCCGTAACGAAGAGATTGCTCAGGTGGTCATTGAGACCGCACAAGAAGCGATGCGCTGGGTTGGAGACCACTGGAACTTCCGGTGTCTTCTGGATACCGAAGGTAAGATGGGTCCTAATTGGGCGATTTGCCACTGATACAGGAGGCTACTCATGAACGAAAGACACTTAACAGGTGCTGCTTCTGAAATGCTAGTAGCCTACAAATTTACCAAAGCTGGGTACACTGTCTATTACCCTATGCTGACTCAGAGTAAAGAGGACTTGGTTGTATG                                                                                                                                                                                                                                                                                                              <  1:25963/301‑1`  
     `GGGTACATGATGAAATCAAAGTAGGCTGCCGTACCGAAGAGATTGCTCAGGTGGTCATTGAGACCGCACAAGAAGCGATGCGCTGGGTTGGAGACCACTGGAACTTCCGGTGTCTTCTGGATACCGAAGGTAAGATGGGTCCTAATTGGGCGATTTGCCACTGATACAGGAGGCTACTCATGAACGAAAGACACTTAACAGGTGCTGCTTCTGAAATGCTAGTAGCCTACAAATTTACCAAAGCTGGGTACACTGTCTATTACCCTATGCTGACTCAGAGTAAAGAGGACTTGGTTGTATG                                                                                                                                                                                                                                                                                                              <  1:106922/301‑1`  
       `GTACATGATGAAATCCAAGTAGGCTGCCGTACCGAAGAGATTGCTCAGGTGGTCATTGAGACCGCACAAGAAGCGATGCGCTGGGTTGGAGACCACTAGAACTTCCGGTGTCTTCTGGATACCGAAGGTAAGATGGGTCCTAATTGGGCGATTTGCCACTGATACAGGAGGCTACTCATGAACGAAAGACACTTAACAGGTGCTGCTTCTTAAATGCTAGTAGCCTACAAATTTACCAAAGCTGGGTACACTGTCTATTACCCTATGCTGACTCAGAGTAAAGAGGACTTGGTTGTATGct                                                                                                                                                                                                                                                                                                            >  1:369666/1‑299`  
          `taagagtggtagcaaggagtgTGCCGTAACGAACAGATTGCTCAGGTGGTCATTGAGACAGGACAAAAAGCGATGCGCTGGGTGTGATACCACTGGAACTTCCGGTGTCTTCTGGATACCGAAGGTAAGATTGGTCCTAATTGGGCGATTTGCCACTGATACAGGAGGCTACTCATTAACGAAAGACACTTAACAGGTGCTGCTTCTGAAATGCTAGTAGCCTACAAATTTACCAAAGCTGGGTACACTGTCTATTACCCTATGCTGACTCAGAGTAAAGAGGACTTGGTTGTATGGAAGG                                                                                                                                                                                                                                                                                                         <  1:284672/280‑1`  
          `CATGATGAACTCCAAGTAGGCTGCCGTACCGAAGAGATTGCTCAGGTGGTCATTGAGACCGCACAAGAAGCGATGCGCTGGGTTGGAGACCACTGGAACTTCCGGTGTCTTCTGGATACCGAAGGTAAGATGGGTCCTAATTGGGCGATTTGCCACTGATACAGGAGGCTACTCATGAACGAAAGACACTTAACAGGTGCTGCTTCTGAAATGCTAGTAGCCTACAAATTTACCAAAGCTGGGTACACTGTCTATTACCCTATGCTGACTCAGAGTAAAGAGGACTTGGTTGctgtctctt                                                                                                                                                                                                                                                                                                         >  1:504683/1‑292`  
              `ATGAAATCCAAGTAGGCTGCCGTACCGAAGAGATTGCTCAGGTGGTCATTGAGACCGCACAAGAAGCGATGCTCTGGGTTGGAGACCACTGGAACTTCCGGTGTCTTCTGGATACCGAAGGTAAGATGGGTCCTAATTGGGCGATTTGCCACTGATACAGGAGGCTAATCATGAACGAAAGACACTTAACAGGTGCTGCTTCTGAAATGCTAGTAGCCTACAAATTTACCAAAGCTGGGTACAATGTCTATTACCCTATGCTGACTCAGAGTAAAGAGGACTTGGTTGTATGctgtcactt                                                                                                                                                                                                                                                                                                     >  1:452664/1‑292`  
                  `AATCCAAGTAGGCTGCCGTACCGAAGAGATTGCTCAGGTGGTCATTGAGACCGCACAAGAAGCGATGCGCTGGGTTGGAGACCACTGGAACTTCCGGTTTCTTCTGGATACCGAAGGTAAGATGGGTCCTAATTGGGCGATTTGCCACTGATACAGGAGGCTACTCATGAACGAAAGACACTTAACAGGTGCTGCTTCTGAAATGCTAGTAGCCTACAAATTTACCAAAGCTGGGTACACTGTCTATTACCCTATGCTGACTCAGAGTAAAGAGGACTTGGTTGTATGctgtctcttataa                                                                                                                                                                                                                                                                                                 >  1:432310/1‑288`  
                                   `GTACCGAAGAGATTGCTCAGGTGGTCATTGAGACCGCACAAGAAGCGATGCGCTGGGTTGGAGACCACTGGAACTTCCGGTGTCTTCTGGATACCGAAGGTAAGATGGGTCCTAATTGGGCGTTTTGCCACTGATACAGGAGGCTACTCATGAACGAAAGACACTTAACAGGTGCTGCTTCTGAAATGCTAGTAGCCTACAAATTTACCAAAGCTGGGTACACTGTCTATTACCCTATGCTGACTCAGAGTAAAGAGGACTTGGTTGTATGctgtctcttatacacatctccgagcccacg                                                                                                                                                                                                                                                                                >  1:148361/1‑271`  
                                                         `GGTCATTGAGACCGCACAAGAAGCGATGCGCTGGGTTGGAGACCACTGGAACTTCCGGTGTCTTCTGGATACCGAAGGTAAGATGGGTCCTAATTGGGCGATTTGCCACTGATACAGGAGGCTACTCATGAACGAAAGACACTTAACAGGTGCTGCTTCTGAAATGCTAGTAGCCTACAAATTTACCAAAGCTGGGTACACTGTCTATTACCCTATGCTGACTCAGAGTAAAGAGGACTTGGTTGTATGGAAGGATTGATGACATTCGTAGTACGTACATCATTTCGTGAGGCCATTAAGG                                                                                                                                                                                                                                                          >  1:410456/1‑301`  
                                                                                                                                                                                                                                                                                                   `taagaggcaTGTATGGAATGATCGATGACATTCATAGTACGTACATCATTTCGTGAGGCCATTAAGGACATGCACGAAGAGTATGCAGATAAGGACTCTTTCAAACAATCTCCTGCAACAGTACGGGAGGTGTTCTGATGTCTGACTACATGAAAGTGCTGCAAGCAATCAAAAGTTGCCCTAAGACTTTCCAGTCCAACTATGTACGGAACAAGGCGAGCCTCGTAGCGGAGGCCGCTTCCCGTGGTCACATCTCGTGCCTGACTACTAGTGGACGTAACGGTGGCGCTTGGGAAATCAC                <  1:325966/292‑1`  
                                                                                                                                                                                                                                                                                                      `gagacaggaATGGAAGGATTGATGACATTCGTAGTACGTACATCATTTCGTGAGGCCATTAAGGACATGCACGAAGAGTATGCAGATAAGGACTCGTTCAAACAATCTCCTGCAACAGTACGGGAGGTGTTATGATGTCTGACTACCGGAAAGTGCTGCAAGCAATCAAAAGTTGCCCTAAGACTTTCCAGTCCAACGATGTACGGAACAATGCGAGCCTCGTAGCGGAGGCCGCTTCCCGTGGTCACATCTCGTGCCTGACTACTAGTGGACGTAACGGTGGCGCTTGGGAAATCACTGC             <  1:286865/292‑1`  
                                                                                                                                                                                                                                                                                                             `GTATGGAATGATTGATGACATTCGTAGTACGTACATCATTTCGTGAGGCCATTAAGGACATGCACGAAGAGTATGCAGATAAGGACTCTTTCAAACAATCTCCTGCAACAGTACGGGAGGTGTTCTGATGTCTGACTACCTGAAAGTGCTGCAAGCAATCAAAAGTTGCCCTAAGACTTTCCAGTCCAACTATGTACGGAACAATTCGAGCCTCGTAGCGGAGGCCGTTTCCCGTGGTCACATCTCGTGCCTGACTACTAGTGGACGTAACGGTGGCGCTTGGGAAATCACTGCTTCCGGT      >  1:255690/1‑301`  
                                                                                                                                                                                                                                                                                                             `GTATGGAAGTATTGATGACATTCGTAGTACGTACATCATTTCGTGAGTCCATTAAGGACATGCACGAAGAGTATGCAGATAAGGACTCTTTCAAACAATCTCCTGCAACAGTACGGGAGGTGTTCTGATGTCTGACTACCTGAAAGTGCTGCAAGCAATCAAAAGTTGCCCTAAGACTTTCCAGTCCAACTATGTACGGAACAATGCGAGCCTCGTAGCGGAGGCCGCTTCCCGTGGTCACATCTCGTGCCTGACTACTAGTGGACGTAACGGTGGCGCTTGGGAAATCACTGCTTCCGGT      >  1:7958/1‑301`  
                                                                                                                                                                                                                                                                                                             `GTATGGAAGTATTGATGACATTCGTAGTACGTACATCATTTCGTGAGGCCATTAAGGACATGCACGAAGAGTATGCAGATAAGGACTCTTTCAAACAATCTCCTGCAACAGTACGGGAGGTGTTCTGATGTCTGACTCCCTGAAAGTGCTGCAAGCAATCAAAAGTTGCCCTAAGACTTTCCAGTCAAACTATGTACGGAACAATGCGAGCCTCGTAGCGGAGGCCGCTTCCCGTGGTCACATCTCGTGCCTGACTACTAGTGGACGTAACGGTGGCGCTTGGGCACTCACctgtctctta      >  1:244237/1‑291`  
                                                                                                                                                                                                                                                                                                             `GTATGGAAGGATTGTTGACATTCGTAGTACGTACATCATTTCGTGAGGCCATTAAGGACATGCACGAAGAGTATGCAGATAAGGACTCTTTCAAACAATCTCCTGCAACAGTACGGGAGGTGTTCTGATGTCTGACTACCTGAAAGTGCTGCAAGCAATCAAAAGTTGCCCTAAGACTTTCCAGTCCAACTATGTACGGAACAATGCGAGCCTCGTAGCGGAGGCCGCTTCCCGTGGTCACATCTCGTGCCTGCCTACTAGTTGACGTAACGGTGGCGCTTTGGAACTCACTGCTTCCGGT      >  1:419665/1‑301`  
                                                                                                                                                                                                                                                                                                             `GTATGGAAGGATTGATTACATTCGTAGTACGTACATCATTTCGTGAGGCCATTAAGGACATGCACGAAGAGTATGCAGATAAGGACTCTTTCAAACAATCTCCTGCAACAGTACGGGAGGTGTTCTGATGTCTGACTACCTGAAAGTGCTGCAAGCAATCAAAAGTTGCCCTAAGACTTTCAAGTCCAACTATGTACGGAACAATGCGAGCCTCGTAGCGGAGGCCGCTTCCCGTGGTCACATCTCGTGCCTGACTACTAGTGGACGTAACGGTGGCGCTTGGGAAATCACTGCTTCCGGT      >  1:508577/1‑301`  
                                                                                                                                                                                                                                                                                                             `GTATGGAAGGATTGATGACATTCGTATTACGTACATCATTTCGTGAGGCCATTAAGGACATGCACGAAGAGTATGCAGATAATGACTCTTTCAAACAATCTCCTGCAACAGTACGGGAGGTGTTCTGCTGTCTGACTACCTGAAAGTACTGCAAGCAATCAAAAGTTGCCCTAAGACTTTCCAGTCCAACTATGTACGGAACAATGCTAGCCTCGTAGCGGAGGCCGCTTCCAGTGGTCACATCTCGTGCCTGACTACTAGTGGACGTAACGGTGGCGCTTGGGAAATCACTGCTTCCGGT      <  1:143350/301‑1`  
                                                                                                                                                                                                                                                                                                             `GTATGGAAGGATTGATGACATTCGTAGTATGTACATCATTTCGTGAGGCCATTAAGGACATGCACGAAGAGTATGCAGATAAGGACTCTTTCAAACAATCTCCTGCAACAGTACGGGAGGTGTTCTGATGTCTGACTACCTGAAAGTGCTGCAAGCAATCATAAGTTGCTCTTAGACTTTCCAGTCCAACTATGTACGGAACAATGCGAGCCTCGTAGCGGAGGCCGCTTCCCGTGGTCACATCTCGTGCCTGACTACTAGTGGACGTAACGGTGGCGCTTGGGAAATCACTGCGTTCGGT      >  1:174858/1‑301`  
                                                                                                                                                                                                                                                                                                             `GTATGGAAGGATTGATGACATTCGTAGTACGTACATCATTTTGTGAGGCCATTAAGGACATGCACGAAGAGTATGCAGATAAGGACTCTTTCAAACAATCTCCTGCAACAGTACGGGAGGTGTTCTGATGTCTGACTACCTGAAAGTGCTGCAAGCAATCAAAAGTTGCCCTAAGACTTTCCAGTCCAACTATGTACGGAACAATGCGAGCCTCGTAGCGGAGGCCGCTTCCCGTGGTCACATCTCGTGCCTGACTACTAGTGGACGTAACGGTGGCGCTGGAGAAATCACTGCTTCCGGT      >  1:112924/1‑301`  
                                                                                                                                                                                                                                                                                                             `GTATGGAAGGATTGATGACATTCGTAGTACGTACATCATTTCGTGAGGCTATTAAGGACATGCACGAAGAGTATGCAGATAAGGACTCTTTCAAACAATCTCCTGCAACAGTACGGGAGGTGTTCTGATGTCTGACTACCTGAAAGTGCTGCAAGCAATCAAAAGTTGCCCTAAGACTTTCCAGTCCAACTATGTACGGAACAATGCGAGCCTCGTAGCGGAGGCCGCTTCCCGTGGTCACATCTCGTGCCTGACTACTAGTGGACGTAACGGTGGCGCTTGGGAAATCACTGCTTCCGGT      >  1:26615/1‑301`  
                                                                                                                                                                                                                                                                                                             `GTATGGAAGGATTGATGACATTCGTAGTACGTACATCATTTCGTGAGGCCATTAAGGACATGCACGAAGAGTATGCAGATAAGGACTCTTTCAAACATTCTCCTGCAACAGTACGGGAGGTGTTCTGATGTCTGACTACCTGAAAGTGCTGCAAGCAATCACAAGTTGCCCTAAGACTTTCCAGTCCAACTATGTACGGAACAATGCGAGCCTCGTAGCGGAGGCCGCTTCCCGTGGTCACATCTCGTGCCTGACTACTAGTGGACGTAACGGTGGCGCTTGGGAAATCACTGCTTCCGGT      >  1:315309/1‑301`  
                                                                                                                                                                                                                                                                                                             `GTATGGAAGGATTGATGACATTCGTAGTACGTACATCATTTCGTGAGGCCATTAAGGACATGCACGAAGAGTATGCAGATAAGGACTCTTTCAAACAATCTCCTTCAACAGTACGGGAGGTGTTCTGATGTCTGACTACCTGAAAGTGCTGCAAGCAATCAAAAGTTGTCCTAAGACTTTCCAGTCCAACTATGTACGGAACAATGCGAGCCTCGTAGCGGAGGCCGATTCCCGTGGTCACATCTCGTGCCTGACTACTAGTGGACGTAACGGTGGCGCTTGGGAAATCACTGCTTCCGGT      >  1:304331/1‑301`  
                                                                                                                                                                                                                                                                                                             `GTATGGAAGGATTGATGACATTCGTAGTACGTACATCATTTCGTGAGGCCATTAAGGACATGCACGAAGAGTATGCAGATAAGGACTCTTTCAAACAATCTCCTGCAACAGTACTGGAGGTGTTCTGATGTCTGACTACCTGAAAGTGCTGCAAGAAATCAAAAGTTGCCCTAAGACTTTCCAGTACAACTATGTACGGAACAATGCGCACCTCGTAGCGGAGGCAGCTTCCAGTGTTCACATCCCGTGCCTGACTACTAGTTGACGTAACGGTGGCTCTTGGGAAATCACTGCTGCCGGT      >  1:301529/1‑301`  
                                                                                                                                                                                                                                                                                                             `GTATGGAAGGATTGATGACATTCGTAGTACGTACATCATTTCGTGAGGCCATTAAGGACATGCACGAAGAGTATGCAGATAAGGACTCTTTCAAACAATCTCCTGCAACAGTACGGTAGGTGTTCTGATGTCTGACTACCTGAAAGTGCTGCAAGCAATCAAAAGTTGCCCTAAGACTTTCCAGTCCAACTATGTACGGAACAATGCGAGCCTCGTAGCGGAGGCCGCTTCCCGTGGTCACATCTCGTGCCTGACTACTAGTGGACGTAACTGTGGCGCTTGGGAAATCACTGCTTCCGGT      >  1:497314/1‑301`  
                                                                                                                                                                                                                                                                                                             `GTATGGAAGGATTGATGACATTCGTAGTACGTACATCATTTCGTGAGGCCATTAAGGACATGCACGAAGAGTATGCAGATAAGGACTCTTTCAAACAATCTCCTGCAACAGTACGGGGGGTGTTCTGATGTCTGACTACCTGAAAGTGCTGCAAGCAATCAAAAGTTGCCCTAAGACTTTCCAGTCCAACTATGTACGGAACAATGCGAGCCTCGTAGCGGAGGCCGCTTCCCGTGGTAACATCTCGTGCCTGACTACTAGTGGACGTAACGGGGGCGCTTGGGAAATCACTGCTTCCGGT      >  1:183756/1‑301`  
                                                                                                                                                                                                                                                                                                             `GTATGGAAGGATTGATGACATTCGTAGTACGTACATCATTTCGTGAGGCCATTAAGGACATGCACGAAGAGTATGCAGATAAGGACTCTTTCAAACAATCTCCTGCAACAGTACGGGAGGTGTTCTGATGTCTGACTCCCTGAAAGTGCTGCAAGCAATCAAAAGTTGCCCTAAGACTTTCCAGTCCAACTATGTACGGAACAATGCGAGCCTCGTAGCGGATGCAGCTTCCCGTGGTCACATATCGTGCCTGACTACTAGTGGACGTAACGGTGGCGCTTGGGAAATCACTGCTTCCGGT      >  1:227837/1‑301`  
                                                                                                                                                                                                                                                                                                             `GTATGGAAGGATTGATGACATTCGTAGTACGTACATCATTTCGTGAGGCCATTAAGGACATGCACGAAGAGTATGCAGATAAGGACTCTTTCAAACAATCTCCTGCAACAGTACGGGAGGTGTTCTGATGTCTGACTATCTGAAAGTGCTGCAAGCAATCAAAAGTTGCCCTAAGACTTTCCAGTCCAACTATGTACGGAACAATGCGAGCCTCGTAGCGGAGGCCGCTTCCCGTGGTCACATCTCGTGCCTGACTACTAGTGGACGTAACGGTGGCGCGTGGGAAATCACTGCTTACGGT      >  1:54983/1‑301`  
                                                                                                                                                                                                                                                                                                             `GTATGGAAGGATTGATGACATTCGTAGTACGTACATCATTTCGTGAGGCCATTAAGGACATGCACGAAGAGTATGCAGATAAGGACTCTTTCAAACAATCTCCTGCAACAGTACGGGAGGTGTTCTGATGTCTGACTACCTGAAAGTGCTGCAAGCAATCAAAAGTTGCCCTAAGACTTTCCAGTCCAACTATGTACGGAACAATGCTAGCCTCGTATCGGAGGCCGCTTCCCGTGGTCACATCTCGTGCCTGACTACTAGTGGACGTAACGGTGGCGCTTGGGAAATCACTGCTTCCGGg      >  1:125013/1‑300`  
                                                                                                                                                                                                                                                                                                             `GTATGGAAGGATTGATGACATTCGTAGTACGTACATCATTTCGTGAGGCCATTAAGGACATGCACGAAGAGTATGCAGATAAGGACTCTTTCAAACAATCTCCTGCAACAGTACGGGAGGTGTTCTGATGTCTGACTACCTGAAAGTGCTGCAAGCAATCAAAAGTTGCCCTAAGACTTTCCAGTCCAACTATGTACGGAACAATGCGAGCCTCGTAGTGGAGGCCGCTTCCCGTGGTCACATCTCGTGCCTGACTACTAGTGGACGTAACGGTGGCGCTTGGGAAATCACTGCTTCCGGT      >  1:25234/1‑301`  
                                                                                                                                                                                                                                                                                                             `GTATGGAAGGATTGATGACATTCGTAGTACGTACATCATTTCGTGAGGCCATTAAGGACATGCACGAAGAGTATGCAGATAAGGACTCTTTCAAACAATCTCCTGCAACAGTACGGGAGGTGTTCTGATGTCTGACTACCTGAAAGTGCTGCAAGCAATCAAAAGTTGCCCTAAGACTTTCCAGTCCAACTATGTACGGAACAATGCGAGCCTCGTAGCGTAGGCCGCTTCCCGTGGTCACATCTCGTGCCTGACTACTAGTGGACGTAACGGTGGCGATTGGGAAATCACTGCTTCCGGT      >  1:223695/1‑301`  
                                                                                                                                                                                                                                                                                                             `GTATGGAAGGATTGATGACATTCGTAGTACGTACATCATTTCGTGAGGCCATTAAGGACATGCACGAAGAGTATGCAGATAAGGACTCTTTCAAACAATCTCCTGCAACAGTACGGGAGGTGTTCTGATGTCTGACTACCTGAAAGTGCTGCAAGCAATCAAAAGTTGCCCTAAGACTTTCCAGTCCAACTATGTACGGAACAATGCGAGCCTCGTAGCGGAGGCTGCTTCCCGTGGTCACATCTCGTGCCTGACTACTAGTGGACGTAACGGTGGCGCTTGGGAAATCACTGCTTCCGGT      <  1:268251/301‑1`  
                                                                                                                                                                                                                                                                                                             `GTATGGAAGGATTGATGACATTCGTAGTACGTACATCATTTCGTGAGGCCATTAAGGACATGCACGAAGAGTATGCAGATAAGGACTCTTTCAAACAATCTCCTGCAACAGTACGGGAGGTGTTCTGATGTCTGACTACCTGAAAGTGCTGCAAGCAATCAAAAGTTGCCCTAAGACTTTCCAGTCCAACTATGTACGGAACAATGCGAGCCTCGTAGCGGAGGCCGCTTCCCGTGGTTACATCTCGTGCCTGACTACTAGTGGACGGTAGGGTGGCGCTGGGGAAATCATTGCTTCCGGT      >  1:248202/1‑301`  
                                                                                                                                                                                                                                                                                                             `GTATGGAAGGATTGATGACATTCGTAGTACGTACATCATTTCGTGAGGCCATTAAGGACATGCACGAAGAGTATGCAGATAAGGACTCTTTCAAACAATCTCCTGCAACAGTACGGGAGGTGTTCTGATGTCTGACTACCTGAAAGTGCTGCAAGCAATCAAAAGTTGCCCTAAGACTTTCCAGTCCAACTATGTACGGAACAATGCGAGCCTCGTAGCGGAGGCCGCTTCCCGTGGTCACATCTCGTGCCTGACTCCTAGTGGACGTAACGGTGGCGCTTGGGAAATCACTGCTTCCGGT      >  1:492740/1‑301`  
                                                                                                                                                                                                                                                                                                             `GTATGGAAGGATTGATGACATTCGTAGTACGTACATCATTTCGTGAGGCCATTAAGGACATGCACGAAGAGTATGCAGATAAGGACTCTTTCAAACAATCTCCTGCAACAGTACGGGAGGTGTTCTGATGTCTGACTACCTGAAAGTGCTGCAAGCAATCAAAAGTTGCCCTAAGACTTTCCAGTCCAACTATGTACGGAACAATGCGAGCCTCGTAGCGGAGGCCGCTTCCCGTGGTCACATCTCGTGCCTGACTACTAGTGGACGTAACGGTGGCGCTTGGGAAATCctgtctcttata      >  1:196455/1‑289`  
                                                                                                                                                                                                                                                                                                             `GTATGGAAGGATTGATGACATTCGTAGTACGTACATCATTTCGTGAGGCCATTAAGGACATGCACGAAGAGTATGCAGATAAGGACTCTTTCAAACAATCTCCTGCAACAGTACGGGAGGTGTTCTGATGTCTGACTACCTGAAAGTGCTGCAAGCAATCAAAAGTTGCCCTAAGACTTTCCAGTCCAACTATGTACGGAACAATGCGAGCCTCGTAGCGGAGGCCGCTTCCCGTGGTCACATCTCGTGCCTGACTACTAGTGGACGTAACGGTGGCGCTTGGGAAATCACTGCTTCCGct      >  1:6808/1‑299`  
                                                                                                                                                                                                                                                                                                             `GTATGGAAGGATTGATGACATTCGTAGTACGTACATCATTTCGTGAGGCCATTAAGGACATGCACGAAGAGTATGCAGATAAGGACTCTTTCAAACAATCTCCTGCAACAGTACGGGAGGTGTTCTGATGTCTGACTACCTGAAAGTGCTGCAAGCAATCAAAAGTTGCCCTAAGACTTTCCAGTCCAACTATGTACGGAACAATGCGAGCCTCGTAGCGGAGGCCGCTTCCCGTGGTCACATCTCGTGCCTGACTACTAGTGGACGTAACGGTGGCGCTTGGGAAATCACTGCTTCCGGT      >  1:312068/1‑301`  
                                                                                                                                                                                                                                                                                                             `GTATGGAAGGATTGATGACATTCGTAGTACGTACATCATTTCGTGAGGCCATTAAGGACATGCACGAAGAGTATGCAGATAAGGACTCTTTCAAACAATCTCCTGCAACAGTACGGGAGGTGTTCTGATGTCTGACTACCTGAAAGTGCTGCAAGCAATCAAAAGTTGCCCTAAGACTTTCCAGTCCAACTATGTACGGAACAATGCGAGCCTCGTAGCGGAGGCCGCTTCCCGTGGTCACATCTCGTGCCTGACTACTAGTGGACGTAACGGTGGCGCTTGGGAAATCACTGCTTCCGGT      >  1:421031/1‑301`  
                                                                                                                                                                                                                                                                                                             `GTATGGAAGGATTGATGACATTCGTAGTACGTACATCATTTCGTGAGGCCATTAAGGACATGCACGAAGAGTATGCAGATAAGGACTCTTTCAAACAATCTCCTGCAACAGTACGGGAGGTGTTCTGATGTCTGACTACCTGAAAGTGCTGCAAGCAATCAAAAGTTGCCCTAAGACTTTCCAGTCCAACTATGTACGGAACAATGCGAGCCTCGTAGCGGAGGCCGCTTCCCGTGGTCACATCTCGTGCCTGACTACTAGTGGACGTAACGGTGGCGCTTGGGAAATCACTGCTTCCGGT      <  1:216166/301‑1`  
                                                                                                                                                                                                                                                                                                             `GTATGGAAGGATTGATGACATTCGTAGTACGTACATCATTTCGTGAGGCCATTAAGGACATGCACGAAGAGTATGCAGATAAGGACTCTTTCAAACAATCTCCTGCAACAGTACGGGAGGTGTTCTGATGTCTGACTACCTGAAAGTGCTGCAAGCAATCAAAAGTTGCCCTAAGACTTTCCAGTCCAACTATGTACGGAACAATGCGAGCCTCGTAGCGGAGGCCGCTTCCCGTGGTCACATCTCGTGCCTGACTACTAGTGGACGTAACGGTGGCGCTTGGGAAATCACTGCTTCCGGT      <  1:208265/301‑1`  
                                                                                                                                                                                                                                                                                                             `GTATGGAAGGATTGATGACATTCGTAGTACGTACATCATTTCGTGAGGCCATTAAGGACATGCACGAAGAGTATGCAGATAAGGACTCTTTCAAACAATCTCCTGCAACAGTACGGGAGGTGTTCTGATGTCTGACTACCTGAAAGTGCTGCAAGCAATCAAAAGTTGCCCTAAGACTTTCCAGTCCAACTATGTACGGAACAATGCGAGCCTCGTAGCGGAGGCCGCTTCCCGTGGTCACATCTCGTGCCTGACTACTAGTGGACGTAACGGTGGCGCTTGGGAAATCACTGCTTCCGGT      >  1:268844/1‑301`  
                                                                                                                                                                                                                                                                                                             `GTATGGAAGGATTGATGACATTCGTAGTACGTACATCATTTCGTGAGGCCATTAAGGACATGCACGAAGAGTATGCAGATAAGGACTCTTTCAAACAATCTCCTGCAACAGTACGGGAGGTGTTCTGATGTCTGACTACCTGAAAGTGCTGCAAGCAATCAAAAGTTGCCCTAAGACTTTCCAGTCCAACTATGTACGGAACAATGCGAGCCTCGTAGCGGAGGCCGCTTCCCGTGGTCACATCTCGTGCCTGACTACTAGTGGACGTAACGGTGGCGCTTGGGAAATCACTGCTTCCGGT      >  1:373354/1‑301`  
                                                                                                                                                                                                                                                                                                             `GTATGGAAGGATTGATGACATTCGTAGTACGTACATCATTTCGTGAGGCCATTAAGGACATGCACGAAGAGTATGCAGATAAGGACTCTTTCAAACAATCTCCTGCAACAGTACGGGAGGTGTTCTGATGTCTGACTACCTGAAAGTGCTGCAAGCAATCAAAAGTTGCCCTAAGACTTTCCAGTCCAACTATGTACGGAACAATGCGAGCCTCGTAGCGGAGGCCGCTTCCCGTGGTCACATCTCGTGCCTGACTACTAGTGGACGTAACGGTGGCGCTTGGGAAATCACTGCTTCCGGT      >  1:30944/1‑301`  
                                                                                                                                                                                                                                                                                                             `GTATGGAAGGATTGATGACATTCGTAGTACGTACATCATTTCGTGAGGCCATTAAGGACATGCACGAAGAGTATGCAGATAAGGACTCTTTCAAACAATCTCCTGCAACAGTACGGGAGGTGTTCTGATGTCTGACTACCTGAAAGTGCTGCAAGCAATCAAAAGTTGCCCTAAGACTTTCCAGTCCAACTATGTACGGAACAATGCGAGCCTCGTAGCGGAGGCCGCTTCCCGTGGTCACATCTCGTGCCTGACTACTAGTGGACGTAACGGTGGCGCTTGGGAAATCACTGCTTCCGGT      >  1:358066/1‑301`  
                                                                                                                                                                                                                                                                                                             `GTATGGAAGGATTGATGACATTCGTAGTACGTACATCATTTCGTGAGGCCATTAAGGACATGCACGAAGAGTATGCAGATAAGGACTCTTTCAAACAATCTCCTGCAACAGTACGGGAGGTGTTCTGATGTCTGACTACCTGAAAGTGCTGCAAGCAATCAAAAGTTGCCCTAAGACTTTCCAGTCCAACTATGTACGGAACAATGCGAGCCTCGTAGCGGAGGCCGCTTCCCGTGGTCACATCTCGTGCCTGACTACTAGTGGACGTAACGGTGGCGCTTGGGAAATCACTGCTTCCGGT      <  1:186800/301‑1`  
                                                                                                                                                                                                                                                                                                             `GTATGGAAGGATTGATGACATTCGTAGTACGTACATCATTTCGTGAGGCCATTAAGGACATGCACGAAGAGTATGCAGATAAGGACTCTTTCAAACAATCTCCTGCAACAGTACGGGAGGTGTTCTGATGTCTGACTACCTGAAAGTGCTGCAAGCAATCAAAAGTTGCCCTAAGACTTTCCAGTCCAACTATGTACGGAACAATGCGAGCCTCGTAGCGGAGGCCGCTTCCCGTGGTCACATCTCGTGCCTGACTAATAGTGGACGTAACGGTGGCGCTTGGGAAATCACTGCTTCCGGT      >  1:11967/1‑301`  
                                                                                                                                                                                                                                                                                                             `GTATGGAAGGATTGATGACATTCGTAGTACGTACATCATTTCGTGAGGCCATTAAGGACATGCACGAAGAGTATGCAGATAAGGACTCTTTCAAACAATCTCCTGCAACAGTACGGGAGGTGTTCTGATGTCTGACTACCTGAAAGTGCTGCAAGCAATCAAAAGTTGCCCTAAGACTTTCCAGTCCAACTATGTACGGAACAATGCGAGCCTCGTAGCGGAGGCCGCTTCCCGTGGTCACATCCCGTGCCTGACTACTAGTGGACGTAACGGTGGCGCTTGGGAAATAACTGCTTCCGGT      >  1:384713/1‑301`  
                                                                                                                                                                                                                                                                                                             `GTATGGAAGGATTGATGACATTCGTAGTACGTACATCATTTCGTGAGGCCATTAAGGACATGCACGAAGAGTATGCAGATAAGGACTCTTTCAAACAATCTCCTGCAACAGTACGGGAGGTGTTCTGATGTCTGACTACCTGAAAGTGCTGCAAGCAATCAAAAGTTGCCCTAAGACTTTCCAGTCCAACTATGTACGGAACAATGCGAGCCTCGTAGCGGAGGCCGCTTCCCGTGGTAACATCTCGTGCCTGACTACTAGTGGACGTAACGGTGGCGCTTGGGAAATCACctgtctcttt      >  1:200249/1‑291`  
                                                                                                                                                                                                                                                                                                             `GTATGGAAGGATTGATGACATTCGTAGTACGTACATCATTTCGTGAGGCCATTAAGGACATGCACGAAGAGTATGCAGATAAGGACTCTTTCAAACAATCTCCTGCAACAGTACGGGAGGTGTTCTGATGTCTGACTACCTGAAAGTGCTGCAAGCAATCAAAAGTTGCCCTAAGACTTTCCAGTCCAACTATGTACGGAACAATGCGAGCCTCGTAGCGGAGGCCGCTTCCCGTAGTCACATCTCGTGCCTGACTAATAGTTGACGTAATGGTGGCGCTTGGGAAATCACTGCTTCCGGT      >  1:90672/1‑301`  
                                                                                                                                                                                                                                                                                                             `GTATGGAAGGATTGATGACATTCGTAGTACGTACATCATTTCGTGAGGCCATTAAGGACATGCACGAAGAGTATGCAGATAAGGACTCTTTCAAACAATCTCCTGCAACAGTACGGGAGGTGTTCTGATGTCTGACTACCTGAAAGTGCTGCAAGCAATCAAAAGTTGCCCTAAGACTTTCCAGTCCAACTATGTACGGAACAATGCGAGCCTCGTAGCGGAGGCCGCTTCCAGTGGTCACATCTCGTGCCTGACTACTAGTGGACGTAACGGTGGCGCTTGGGAAATCACTGCTTCCGGT      >  1:76978/1‑301`  
                                                                                                                                                                                                                                                                                                             `GTATGGAAGGATTGATGACATTCGTAGTACGTACATCATTTCGTGAGGCCATTAAGGACATGCACGAAGAGTATGCAGATAAGGACTCTTTCAAACAATCTCCTGCAACAGTACGGGAGGTGTTCTGATGTCTGACTACCTGAAAGTGCTGCAAGCAATCAAAAGTTGCCCTAAGACTTTCCAGTCCAACTATGTACGGAACAATGCGAGCCTCGTAGCGGAGGCCGCTTCCAGTGGTCACATCTCGTGACTGACTACTAGTGGACGTAACGGTGGCGCTTGGGAAATCACTGCTTCCGGT      >  1:296436/1‑301`  
                                                                                                                                                                                                                                                                                                             `GTATGGAAGGATTGATGACATTCGTAGTACGTACATCATTTCGTGAGGCCATTAAGGACATGCACGAAGAGTATGCAGATAAGGACTCTTTCAAACAATCTCCTGCAACAGTACGGGAGGTGTTCTGATGTCTGACTACCTGAAAGTGCTGCAAGCAATCAAAAGTTGCCCTAAGACTTTCCAGTCCAACTATGTACGGAACAATGCGAGCCTCGTAGCGGAGGCCGCTTACCGTGGTCACATCTCGTGCCTGACTACTAGTTTACGTAACGGTGGCGCTGGGGAAATAACTGCTTCCGGT      >  1:50735/1‑301`  
                                                                                                                                                                                                                                                                                                             `GTATGGAAGGATTGATGACATTCGTAGTACGTACATCATTTCGTGAGGCCATTAAGGACATGCACGAAGAGTATGCAGATAAGGACTCTTTCAAACAATCTCCTGCAACAGTACGGGAGGTGTTCTGATGTCTGACTACCTGAAAGTGCTGCAAGCAATCAAAAGTTGCCCTAAGACTTTCCAGTCCAACTATGTACGGAACAATGCGAGCCTCGTAGCGGAGGCAGCTTCCCGTGGTCACATCTCGTGCCTGACTACTAGTGGACGTAACGGTGGCGCTTGGGAAATCACTGCTTCCGGT      >  1:430878/1‑301`  
                                                                                                                                                                                                                                                                                                             `GTATGGAAGGATTGATGACATTCGTAGTACGTACATCATTTCGTGAGGCCATTAAGGACATGCACGAAGAGTATGCAGATAAGGACTCTTTCAAACAATCTCCTGCAACAGTACGGGAGGTGTTCTGATGTCTGACTACCTGAAAGTGCTGCAAGCAATCAAAAGTTGCCCTAAGACTTTCCAGTCCAACTATGTACGGAACAATGCGAGCCTCGTAGCGGAGGACGCTTCCCGTGGTCACATCTCGTGCCTGACTACTAGTGGACGTAACGGTGGCGCTTGGGAAATCACTGCTTCCGGT      >  1:96984/1‑301`  
                                                                                                                                                                                                                                                                                                             `GTATGGAAGGATTGATGACATTCGTAGTACGTACATCATTTCGTGAGGCCATTAAGGACATGCACGAAGAGTATGCAGATAAGGACTCTTTCAAACAATCTCCTGCAACAGTACGGGAGGTGTTCTGATGTCTGACTACCTGAAAGTGCTGCAAGCAATCAAAAGTTGCCCTAAGACTTTCCAGTCCAACTATGTACGGAACAATGAGAGCCTCGTAGCGGAGGCCGCTTCCCGTGGTCACATCTCGTGCCTGACTACTAGTGGACGTAACGGTGGCGCTTGGGAAATCACTGCTTCCGGT      >  1:435019/1‑301`  
                                                                                                                                                                                                                                                                                                             `GTATGGAAGGATTGATGACATTCGTAGTACGTACATCATTTCGTGAGGCCATTAAGGACATGCACGAAGAGTATGCAGATAAGGACTCTTTCAAACAATCTCCTGCAACAGTACGGGAGGTGTTCTGATGTCTGACTACCTGAAAGTGCTGCAAGCAATCAAAAGTTGCCCTAAGACTTCCCAGTCCAACTATGTACGGAACAATGCGAGCCTCGTAGCGGAGGCCGCTTCCCGTGGTCACATCTCGTGCCTGACTACTAGTGGACGTAACGGTGGCGCTTGGGAAATCACTGCTTCCGGT      >  1:281716/1‑301`  
                                                                                                                                                                                                                                                                                                             `GTATGGAAGGATTGATGACATTCGTAGTACGTACATCATTTCGTGAGGCCATTAAGGACATGCACGAAGAGTATGCAGATAAGGACTCTTTCAAACAATCTCCTGCAACAGTACGGGAGGTGTTCTGATGTCTGACTACCTGAAAGTGCTGCAAGCAATCAAAAGTTGCCATAAGACTTTCCAGTCCAACTATGTACGGAACAATGCGAGCCTCGTAGCGGAGGCCGCTTCCCGTGGTCACATCTCGTGCCTGACTACTAGTGGACGTAACGGTGGCGCTTGGGAAATCACTGCTTCCGGT      >  1:282187/1‑301`  
                                                                                                                                                                                                                                                                                                             `GTATGGAAGGATTGATGACATTCGTAGTACGTACATCATTTCGTGAGGCCATTAAGGACATGCACGAAGAGTATGCAGATAAGGACTCTTTCAAACAATCTCCTGCAACAGTACGGGAGGTGTTCTGATGTCTGACTACCTGAAAGTGCTGCAAGCAATCAAAAGTTGCCATAAGACTTTCCAGTCCAACTATGTACGGAACAATGCGAGCCTCGTAGCGGAGGCCGCTTCCCGTGGTCACATCTCGTGCCTGACTACTAGTGGACGTAACGGTGGCGCTTGGGAAAACACTGCTTCCGGT      >  1:468324/1‑301`  
                                                                                                                                                                                                                                                                                                             `GTATGGAAGGATTGATGACATTCGTAGTACGTACATCATTTCGTGAGGCCATTAAGGACATGCACGAAGAGTATGCAGATAAGGACTCTTTCAAACAATCTCCTGCAACAGTACGGGAGGTGTTCTGATGTCTGACTACCTGAAAGTGCTGCAAGCAATCAAAAGTTGCCATAAGACTTTCCAGTCCAACTATGTACGGAACAATGCGAGCCTCGTAGAGGAGGCCGCTTCCCGTGGTCACATCTCGTGCCTGACTACTAGTGGACGTAACGGTGGCGCTTGGGAAATCACTGCTTCCGGT      >  1:273022/1‑301`  
                                                                                                                                                                                                                                                                                                             `GTATGGAAGGATTGATGACATTCGTAGTACGTACATCATTTCGTGAGGCCATTAAGGACATGCACGAAGAGTATGCAGATAAGGACTCTTTCAAACAATCTCCTGCAACAGTACGGGAGGTGTTCTGATGTCTGACTACCTGAAAGTGCTGCAAGCAATCAAAAGTTGCCATAAGACTTTCCAGTCCAACTATGTACGGAACAATGCGAGCCTAGTAGCGGAGGCCGCTTCCCGTGGTCACATCTCGTGCCTGACTACTAGTGGACGTAACGGGGGCGCTCGGGAAATCACTGCTTACGag      >  1:449938/1‑299`  
                                                                                                                                                                                                                                                                                                             `GTATGGAAGGATTGATGACATTCGTAGTACGTACATCATTTCGTGAGGCCATTAAGGACATGCACGAAGAGTATGCAGATAAGGACTCTTTCAAACAATCTCCTGCAACAGTACGGGAGGTGTTCTGATGTCTGACTACCTGAAAGTGCTGCAAGCAATCAAAAGTTGCACTAAGACTTTCCAGTCCAACTATGTACGGAACAATGCGAGCCTCGTAGCGGAGGCCGCTTCCCGTGGTCACATCTCGTGCCTGACTACTAGTGGACGTAACGGTGGCGCTTGGGAAATCACTGCTTCCGGT      >  1:347820/1‑301`  
                                                                                                                                                                                                                                                                                                             `GTATGGAAGGATTGATGACATTCGTAGTACGTACATCATTTCGTGAGGCCATTAAGGACATGCACGAAGAGTATGCAGATAAGGACTCTTTCAAACAATCTCCTGCAACAGTACGGGAGGTGTTCTGATGTCTGACTACCTGAAAGTCCTGCAAGCAATCAAAAGTTGCCCTAAGACTTTCCAGTCCAACTATGTACGGAACAATGCGAGCCTCGTAGCGGAGGCCGCTTCCCGTGGTCACATCTCGTGCCTGACTACTAGTGGACGTAACGGTGGCGCTTGGGAAATCACTGCTTCCGGT      >  1:317767/1‑301`  
                                                                                                                                                                                                                                                                                                             `GTATGGAAGGATTGATGACATTCGTAGTACGTACATCATTTCGTGAGGCCATTAAGGACATGCACGAAGAGTATGCAGATAAGGACTCTTTCAAACAATCTCCTGCAACAGTACGGGAGGTGTTCTGATGTCTAACTACCTGAAAGTGCTGCAAGCAATCAAAAGTTGCCCTAAGACTTTCCAGTCCAAATATGTACGGAACAATGCGAGCCTCGTAGCGGAGGCCGCTTCCCGTGGTCACATCTCGTGCCTGACTACTAGTTGACGTAACGGTGGCGCTTGGGAAATCACctgcatctta      >  1:94912/1‑291`  
                                                                                                                                                                                                                                                                                                             `GTATGGAAGGATTGATGACATTCGTAGTACGTACATCATTTCGTGAGGCCATTAAGGACATGCACGAAGAGTATGCAGATAAGGACTCTTTCAAACAATCTCCTGCAACAGTACGGGAGGTGTTATGATGTCTGACTACCTGAAAGTGCTGCAAGCAATCAAAAGTTGCCCTAAGACTTTCCAGTCCAACTATGTACGGAACAATGCGAGCCTCGTAGCGGAGGCCGCTTCCCGTGGTCACATCTCGTGCCTGACTACTAGTGGACGTAACGGTGGCGCTTGGGAAATCACTGCTTCCGGT      >  1:47615/1‑301`  
                                                                                                                                                                                                                                                                                                             `GTATGGAAGGATTGATGACATTCGTAGTACGTACATCATTTCGTGAGGCCATTAAGGACATGCACGAAGAGAATGCAGATAATGACTCTTTCAAACAATCTCCTGCAACAGTACGGGAGGTGTTCTGATGTCTGACTACCTGAAAGTGCTGCAAGCAATCAAAAGTTGCCCTAAGACTTTCCAGTCCAACTATGTACGGAACAATGCGAGCCTCGTAGCGGAGGCCGCTTCCCGTGGTCACATCTCGTGCCTGACTACTAGTGGACGTAACGGTGGCGCTTGGGACATCACTGCTTCCGGT      >  1:351720/1‑301`  
                                                                                                                                                                                                                                                                                                             `GTATGGAAGGATTGATGACATTCGTAGTACGTACATCATTTCGTGAGGCCATTAAGGACATGAACGAAGAGTATGCAGATAAGGACTCTTTCAAACAATCTCCTGCAACAGTACGGGAGGTGTTCTGATGTCTGACTACCTGAAAGTGCTGCAAGCAATCAAAAGTTGCCCTAAGACTTTCCAGTCCAACTATGTACGGAACAATGCGAGCCTCGTAGCGGAGGCCGCTTCCCGTGGTCACATCTCGTGCCTGACTACTAGTGGACGTAACGGTGGCGATTGGGAAATCACTGCTTCCGGT      <  1:270651/301‑1`  
                                                                                                                                                                                                                                                                                                             `GTATGGAAGGATTGATGACATTAGTAGTACGTACATCATTTCGTGAGGCCATTAAGGACATGCACGAAGAGTATGCAGATAAGGACTCTTTCAAACAATCTCCTGCAACAGTACGGGAGGTGTTCTGATGTCTGACTACCTGAAAGTGCTGCAAGCAATCAAAAGTTGCCCTAAGACTTTCCAGTCCAACTATGTACTGAACAATGCGAGCCTCGTAGCGGAGGCCGCTTCCCGTGGTCACATCTCGTGCCTGACTAATAGTGGACGTCACGGTGGAGCTTGGGAAATCACTGCTTCCGGT      >  1:257084/1‑301`  
                                                                                                                                                                                                                                                                                                             `GTATGGAAGGATTGATGACATTAGTAGTACGTACATCATTTCGTGAGGCCATTAAGGACATGCACGAAGAGTATGCAGATAAGGACTCTTTCAAACAATCTCCTGCAACAGTACGGGAGGTGTTCTGATGTCTGACTACCTGAAAGTGCTGCAAGCAATCAAAAGTTGCCCTAAGACTTTCCAGTCCAACTATGTACGGAACAATGCGAGCCTCGTAGCGGAGGCCGCTTCCCGTGGTCACATCTCGTGCCTGACTACTAGTGGACGTAACGGTGGCGCTTGGGAAATCACTGATTCCGGT      <  1:472748/301‑1`  
                                                                                                                                                                                                                                                                                                             `GTATGGAAGGAGTGATGACATTCGTAGTACGTACATCATTTCTGGAGGCCATTAAGGACATGCACGAAGAGTATGCAGATAAGTACTCTTTCAAACAATCTCCTGCAACAGTACGGGAGGTGTTCTGATGTCTGACTACCTGAAAGTGCTGCAAGCAATCAAAAGTTGCCCTAAGACTTTCCAGTCCAACTATGTACGGAACAATGCGAGCCTCGTAGCGGAGGCCGCTTCCCGTGGTCACATCTCGTGCCTGACTACTTGTGGACGTAACGGTGGCGCTTGGGAAATCACTGCTTCCGGT      <  1:313914/301‑1`  
                                                                                                                                                                                                                                                                                                              `TATGGAAGGATTGATGACATTCGTAGTACGTACATCATTTCGTGAGGCCATTAAGGACATGCACGAAGAGTATGCAGATAAGGACTCTTTCAAACAATCTCCTGCAACAGTACGGGGGGTGTTCTGATGTCTGACTACCTGAAAGTGCTGCAAGCAATCAAAAGTTGCCCTAAGACTTTCCAGTCCAACTATGTACGGAACAATGCGAGCCTCGTAGCGGAGGCCGCTTCCCGTGGTCACATCTCGTGCCTGACTACTAGTGGACGTAACGGTGGCGCTTGGGAAATCACTGCTTCCGGTA     <  1:400636/301‑1`  
                                                                                                                                                                                                                                                                                                              `TATGGAAGGATTGATGACATTCGTAGTACGTACATCATTTCGTGAGGCCATTAAGGACATGCACGAAGAGTATGCAGATAAGGACTCTTTCAAACAATCTCCTGCAACAGTACGGGATGTGTTCTGATGTCTGACTACCTGAAAGTGCTGCAAGCAATCAAAAGTTGCCCTAAGACTTTCCAGTCCAACTATGTACGGAACAATGCGAGCCTCGTAGCGGAGGCCGCTTCCCGTGGTCACATCTCGTGCCTGACTACTAGTGGACGTAACGGTGGCGCTTGGGAAATCACTGCTTCCGGTA     <  1:219739/301‑1`  
                                                                                                                                                                                                                                                                                                               `agGGAAGGATTGATTACATTCGTAGTACGTACATCATTTCGGGATGCCAGTAAGGACATGCAAGAAGAGGATGCAGATAAGGACTCTTTCAAACAATCTCCTGCAACAGTACGGGAGGTGTTCTGATGTCTGACTACCTGAAAGTGCTGCAAGAAATCAAAAGTTGCCCTAAGACTTGCCAGTCCAACTATGTACGGAACAATGCGAGCCTCGTAGCGGAGGCCGCTTCCCGTGGTCACATCTCGTGCCTTACTACTAGTGGACGTAACGGTGGCGCTTGGGAAATCACTGCTTCCGGTAC    <  1:83384/299‑1`  
                                                                                                                                                                                                                                                                                                               `ATGGAATGATTGATGACATTCGTAGTACGTACATCATTTCGTGAGGCCATTAAGGACATGCACGAAGAGTATGCAGATAATGACTCTTTCAAACAATCTCCTGCAACAGTACGGGAGGTGTTCTGATGTCTGACTACCTGAAAGTGCTTCAAGCAATCAAAAGTTGCCCTAAGACTTTCCAGTCCAACTATGTACGGAACAATGCGAGCCTCGTAGCGGAGGCCGCTTCCCGTGGTTACATCTCGTGCCTGACTACTAGTGGACGTAACGGTGGCGCTTGGGAAATCACTGCTTCCGGTAC    <  1:64658/301‑1`  
                                                                                                                                                                                                                                                                                                               `ATGGAAGGATTGATGACATTCGTAGTACGTACATCATTTCGTGAGGCCATTAAGGACATGCACGAAGAGTATGCAGATAAGGACTCTTTCAAACAATCTCCTGCAACAGTACTGGAGGTGTTCTGATGTCTGACTACCTGAAAGTGCTGCAAGCAATCAAAAGTTGACCTAAGACTTTCCAGTCCAACTATGTACGGAACAATTCGAGCCTCGTAGCGGAGGCCGCTTCCCGTGGTCACACCTCGTGCCTGACTACTAGTGGACGTAACGGTGGCGCTTGGGAAATCACTGCTTCCGGTAC    >  1:324157/1‑301`  
                                                                                                                                                                                                                                                                                                               `ATGGAAGGATTGATGACATTCGTAGTACGTACATCATTTCGTGAGGCCATTAAGGACATGCACGAAGAGTATGCAGATAAGGACTCTTTCAAACAATCTCCTGCAACAGTACGGGAGGTGTTCTGATGTCTGACTACCTGAAAGTGCTGCAAGCAATCAAAAGTTGCCCTAAGACTTTCCAGTCCAACTATGTACGGAACAATGCGAGCCTCGTAGCGGAGGCTGCTTCCCGTGGTCACATCTCGTGCCTGACTACTAGTGGACGTAACGGctgtctcttatacccatctacgagcccacg    >  1:371147/1‑271`  
                                                                                                                                                                                                                                                                                                               `ATGGAAGGATTGATGACATTCGTAGTACGTACATCATTTCGTGAGGCCATTAAGGACATGCACGAAGAGTATGCAGATAAGGACTCTTTCAAACAATCTCCTGCAACAGTACGGGAGGTGTTCTGATGTCTGACTACCCGAAAGTGCTGCAAGCAATCAAAAGTTGCCCTAAGACTTTCCAGTCCAACTATGTACGGAACAATGCGAGCCTCGTAGCGGAGGCCGCTTCCCGTGGTCACATCTCGTGCCTGACTACTAGTGGACGTAACGGTGGCGCTTGGGAAATCACTGCTTCCGGTct    >  1:344820/1‑299`  
                                                                                                                                                                                                                                                                                                               `ATGGAAGGATTGATGACATTCGTAGTACGTACATCATTTCATGAGGCCGTTAAGGACATGCACGAAGAGTATGCAGATAAGGACTCTTTCCAACAATCTCCTGCAACAGTACGGGAGGTGTTCTGCTGTCTGACTACCTGAAAGTGCAGCAAGCAATCAAAAGTTGCCCTAAGACTTTCCAGTCCAACTATGTACGTAACAATGCGAGCCTCGTAGCGGAGGCCGCTTCCCGTGGTCACATCTCGTGCCTGACTACTAGTGGACGTAACGGTGGCGCTTGGGAAATCACTGCTTCCGGTAC    <  1:306900/301‑1`  
                                                                                                                                                                                                                                                                                                               `ATGGAAGGATTGATGACATTCGTAGTAAGTAAAGCATTTCGTGAGGCCATTAAGGACATGCACGAAGAGTATGCAGATAAGGACTCTTTCAAACAATCTCCTGCAACAGTACGGGAGGTGTGCTGATGTCTGACTACCTGAAAGGGCTGCAAGCAATCAAAAGTTGCCCTAAGACTTTCCAGGCCAACTATGTACGGAACAATGCGAGCCTCGTAGCGGAGGCCGCTTCCCGTGGTCACATCTCGTGCCTGACTACTAGTGGACGTAACGGTGGCGCTTGGGAAATCACTGCTTCCGGTAC    <  1:203007/301‑1`  

                                                                                                                                                                                                                                                                                                                                                                                                                                                                                                                                                                                                                                
`GGCATGGGTACATGATGAAATCCAAGTAGGCTGCCGTACCGAAGAGATTGCTCAGGTGGTCATTGAGACCGCACAAGAAGCGATGCGCTGGGTTGGAGACCACTGGAACTTCCGGTGTCTTCTGGATACCGAAGGTAAGATGGGTCCTAATTGGGCGATTTGCCACTGATACAGGAGGCTACTCATGAACGAAAGACACTTAACAGGTGCTGCTTCTGAAATGCTAGTAGCCTACAAATTTACCAAAGCTGGGTACACTGTCTATTACCCTATGCTGACTCAGAGTAAAGAGGACTTGGTTGTATG‑‑‑‑‑‑‑‑‑‑‑‑‑‑‑‑‑‑‑‑‑‑‑‑‑‑‑‑‑‑‑‑‑‑‑‑‑‑‑‑‑‑‑‑‑‑‑‑‑‑‑‑‑‑‑‑‑‑‑‑‑‑‑‑‑‑‑‑‑‑‑‑‑‑‑‑‑‑‑‑‑‑‑‑‑‑‑‑‑‑‑‑‑‑‑‑‑‑‑‑‑‑‑‑‑‑‑‑‑‑‑‑‑‑‑‑‑‑‑‑‑‑‑‑‑‑‑‑‑‑‑‑‑‑‑‑‑‑‑‑‑‑‑‑‑‑‑‑‑‑‑‑‑‑‑‑‑‑‑‑‑‑‑‑‑‑‑‑‑‑‑‑‑‑‑‑‑‑‑‑‑‑‑‑‑‑‑‑‑‑‑‑‑‑‑‑‑‑‑‑‑‑‑‑‑‑‑‑‑‑‑‑‑‑‑‑‑‑‑‑‑‑‑‑‑‑‑‑‑‑‑‑‑‑‑‑‑‑‑‑‑‑‑‑‑‑‑‑‑‑‑‑‑‑‑‑‑‑‑‑‑‑‑‑‑‑‑‑‑‑‑‑‑‑‑‑‑‑‑‑‑‑‑‑‑‑‑‑‑‑‑‑‑‑‑‑‑‑‑‑  >  NC_001604.1/16299‑16604`  
`‑‑‑‑‑‑‑‑‑‑‑‑‑‑‑‑‑‑‑‑‑‑‑‑‑‑‑‑‑‑‑‑‑‑‑‑‑‑‑‑‑‑‑‑‑‑‑‑‑‑‑‑‑‑‑‑‑‑‑‑‑‑‑‑‑‑‑‑‑‑‑‑‑‑‑‑‑‑‑‑‑‑‑‑‑‑‑‑‑‑‑‑‑‑‑‑‑‑‑‑‑‑‑‑‑‑‑‑‑‑‑‑‑‑‑‑‑‑‑‑‑‑‑‑‑‑‑‑‑‑‑‑‑‑‑‑‑‑‑‑‑‑‑‑‑‑‑‑‑‑‑‑‑‑‑‑‑‑‑‑‑‑‑‑‑‑‑‑‑‑‑‑‑‑‑‑‑‑‑‑‑‑‑‑‑‑‑‑‑‑‑‑‑‑‑‑‑‑‑‑‑‑‑‑‑‑‑‑‑‑‑‑‑‑‑‑‑‑‑‑‑‑‑‑‑‑‑‑‑‑‑‑‑‑‑‑‑‑‑‑‑‑‑‑‑‑‑‑‑‑‑‑‑‑‑‑‑‑‑‑‑‑‑‑‑‑‑‑‑‑‑‑‑‑‑‑‑‑‑‑‑‑‑‑‑‑‑‑‑‑‑‑‑‑‑‑‑‑‑‑‑gtatgGAAGGATTGATGACATTCGTAGTACGTACATCATTTCGTGAGGCCATTAAGGACATGCACGAAGAGTATGCAGATAAGGACTCTTTCAAACAATCTCCTGCAACAGTACGGGAGGTGTTCTGATGTCTGACTACCTGAAAGTGCTGCAAGCAATCAAAAGTTGCCCTAAGACTTTCCAGTCCAACTATGTACGGAACAATGCGAGCCTCGTAGCGGAGGCCGCTTCCCGTGGTCACATCTCGTGCCTGACTACTAGTGGACGTAACGGTGGCGCTTGGGAAATCACTGCTTCCGGTACTC  >  NC_001604.1/17028‑17327`

  

Alignment Legend

Aligned base mismatch/match (shaded by quality score): `ATCG/ATCG` `< 3 ≤` `ATCG/ATCG` `< 11 ≤` `ATCG/ATCG` `< 23 ≤` `ATCG/ATCG` `< 34 ≤` `ATCG/ATCG` `< 38 ≤` `ATCG/ATCG`

Unaligned base: `atcg`    Masked matching base: `atcg`    Alignment gap: `‑`    Deleted base: `‑`

Reads not counted as support for junction

read\_name Not counted due to insufficient overlap past the breakpoint.

read\_name Not counted due to not crossing MOB target site duplication.
